# Improving postoperative outcomes in a Brazilian hospital through educational programs based on reports of an international database in cardiac surgery

**DOI:** 10.1186/cc14716

**Published:** 2015-09-28

**Authors:** Pedro Gabriel M de BE Silva, Antonio Baruzzi, Denise Ramos, Giuliano Generoso, Jose Teixeira, Marcelo Jamus, Mariana Okada, Nilza Lasta, Thiago A Macedo, Valter Furlan

**Affiliations:** 1 Hospital Totalcor, Cerqueira Cesar, São Paulo, SP, Brazil

## Introduction

Multicenter databases are useful tools for quality improvement programs. Most of this evidence is based on studies in North America and Europe and little is known in other regions. Since 2011, a Brazilian private cardiovascular center has joined an international registry of cardiac surgeries.

## Objective

To evaluate changes in quality indicators and clinical outcomes of cardiac surgery patients after a multifaceted educational program based on reports of an international database.

## Methods

A multifaceted and continuous educational program based on trimestral reports from the international database was implemented in a Brazilian cardiovascular center. A local team targeted reductions in the time of mechanical ventilation (MV), in length of stay and in the number of inappropriate transfusions. A pilot protocol for rational use of blood products based on guidelines was developed in 2011 [1]. Standard criteria for sedation and extubation in the perioperative period were implemented in 2012. The best hospitals of the database were used as a benchmark to define goals with the surgical and clinical staff. All patients submitted to coronary artery bypass graft (CABG) surgeries were included in the analysis which compared pre and post program in order to observe the impact of the educational intervention.

## Results

From January 2012 to December 2013, 667 CABGs were performed. The predicted risk of in-hospital mortality by the score of the Society of Thoracic Surgeons (validated in the hospital [2]) was 1.2 % in 2012 and 0.96 % in 2013. As shown in Table 1, there was a reduction in transfusion comparing 2012 and 2013. The time in MV and the postoperative length of stay reduced in 2013. Mortality did not increase with an earlier extubation and discharge.

**Table 1:** 

**CABG**	**2012 (*n *= 368)**	**2013 (*n *= 299)**	***p *value**
% of CABGs using blood transfusion	48.7	37	0.005
Mean time for extubation (hours)	11.3	4.3	<0.001
ICU mean length of stay (hours)	64.8	50.4	0.001
Mean PO length of stay (days)	6.5	6	NS
Hospital discharge on the fourth PO day (%)	12.2	33.8	<0.001
ICU readmission (%)	4.6	1.6	0.048
Hospital readmission <30 days (%)	13	4.7	<0.001
30-day mortality (%)	2.17	1 %	0.36

## Conclusion

These results indicate that quality improvement program based on international database reports can improve outcomes in a Brazilian private hospital. Global registries can be useful tools to overcome gaps in clinical practice in different countries.
